# Supplementary material for: The Prostaglandin E2 Receptor EP4 Promotes Vascular Neointimal Hyperplasia through Translational Control of Tenascin C via the cAPM/PKA/mTORC1/rpS6 Pathway
Source: Cells. 2022 Aug 31;11(17):2720. doi: 10.3390/cells11172720 (PMC9454981; doi:10.3390/cells11172720)
Supplement: Supplementary file 1 [file cells-11-02720-s001.zip › cells-1871935-supplementary.pdf]

Supplemental Information

The prostaglandin E2 receptor EP4 promotes vascular neointimal hyperplasia through translational control of tenascin C via the cAPM/PKA/mTORC1/rpS6 pathway

Hu Xu[1†], Bingying Fang[1†], Chengzhen Bao[1], Xiuhui Mao[1], Chunhua Zhu[1], Lan Ye[1], Qian Liu[1], Yaqing Li[1], Chunxiu Du[1], Hang Qi[1], Xiaoyan Zhang[2*], Youfei Guan[1*].

[1]Advanced Institute for Medical Sciences, Dalian Medical University, Dalian 11644, China.

[2]Health Science Center, East China Normal University, Shanghai 200241, China

[*] Corresponding Authors: Prof. Youfei Guan, the Advanced Institute for Medical Sciences, Dalian Medical University, Dalian, 116044, China. E-mail: guanyf@dmu.edu.cn ; Prof. Xiaoyan Zhang, Health Science Center, East China Normal University, Shanghai 200241, China. E-mail: xyzhang@hsc.ecnu.edu.cn

[†] These authors contributed equally to this work.

Running title：VSMC EP4 promotes vascular restenosis

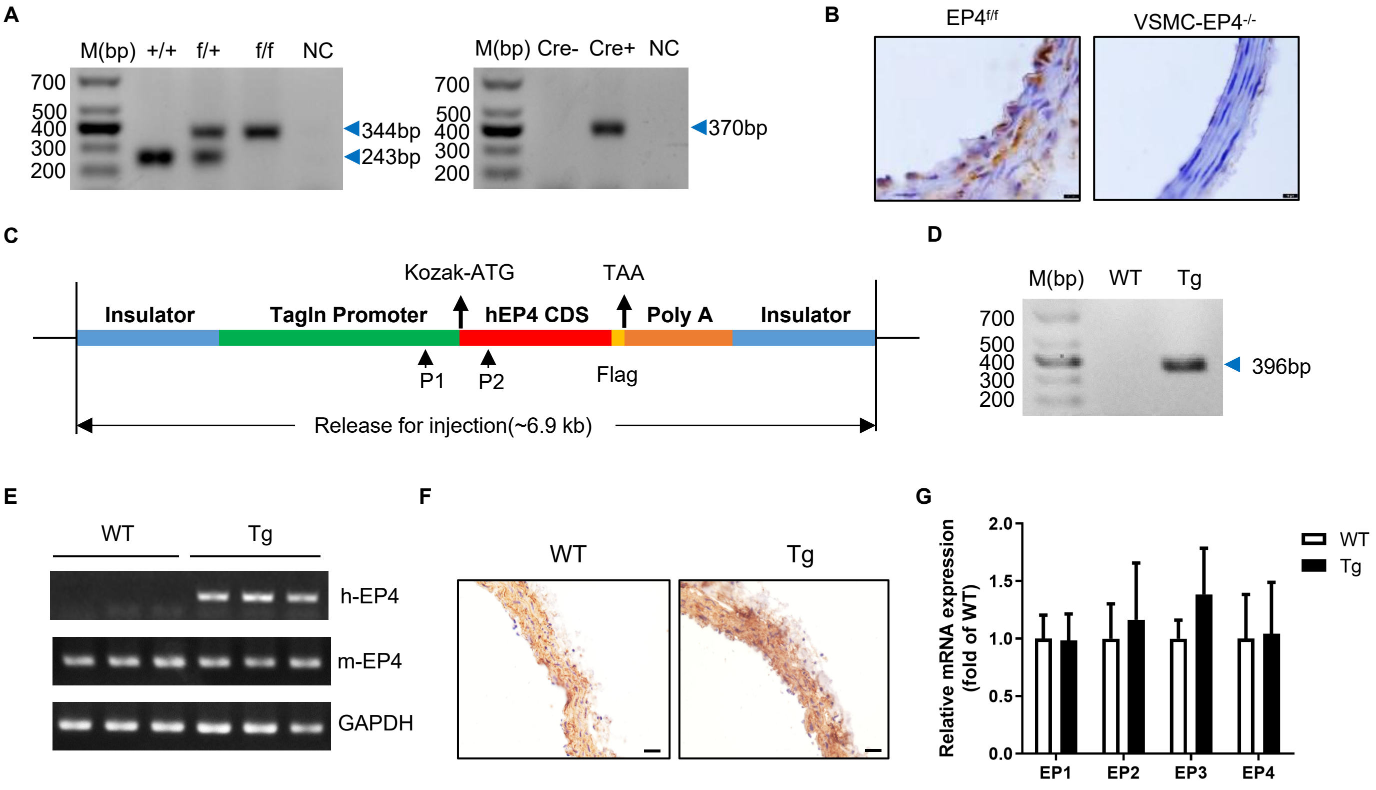

Figure S1. Construction and validation of the VSMC-EP4$^{-/-}$ and VSMC-hEP4 Tg mice

A. PCR validation of the EP4$^{f/f}$, EP4f$^{/+}$ and EP4$^{+/+}$ (wild type, WT) alleles and the Cre recombinase transgene. The 243 bp and 344 bp bands represent the WT allele and the floxed EP4 allele, respectively. The 370 bp band represents the Cre transgene that presents only in the VSMC-EP4$^{-/-}$ mice. M: marker.

B. EP4 immunohistochemical staining showing a prominent reduction of EP4 in the aortic smooth muscle layer of the VSMC-EP4$^{-/-}$ mice. Scale bar = 10 μm.

C. Schematic showing of the generation of VSMC-hEP4-Tg mice (Tg). Human EP4 coding sequence is placed behind the tagln promoter which is used to drive transgene expression in smooth muscle cells. The P1 and P2 are the primers that designed to detect the transgene.

D. PCR validation of the WT and Tg alleles. The band of 396 bp represents Tg positive allele.

E. Human EP4 mRNA expression in the aortas of the WT and Tg mice. The aorta RNA was extracted, and then human and mouse EP4 primers were used to amplify the products by RT-PCR. The PCR products were validated by electrophoresis on 1% agarose.

F. Immunohistochemistry showing the overexpression of EP4 in the aortic smooth muscle layer of the VSMC-hEP4-Tg mice. Scale bar = 10 μm.

G. RT-PCR analysis showing no change in mRNA levels of mouse EP1, EP2, EP3, EP4 receptors in the aortas between WT and VSMC-hEP4-Tg mice. n = 4-9.

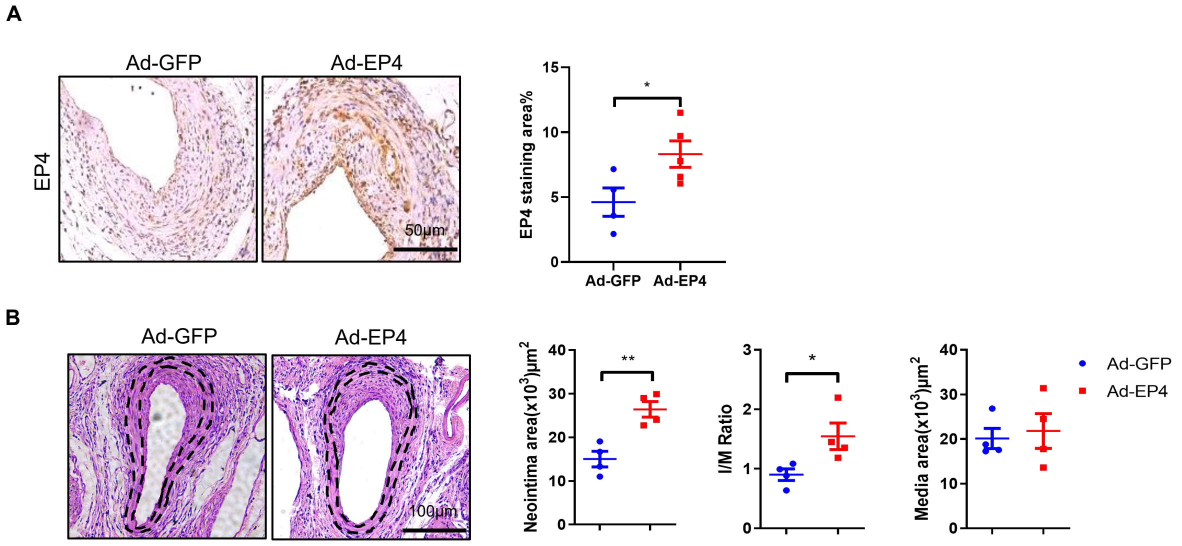

Figure S2. Adenovirus-mediated overexpression of human EP4 enhances neointimal hyperplasia.

A. Mice were subjected to femoral artery wire injury and infected with the Ad-GFP or Ad-EP4 for 14 days. The expression of EP4 was examined by immunohistochemistry staining. Scale bar = 50 μm. n = 4 per group.

B. Representative H&E staining of the femoral arteries in mice infected with the Ad-GFP or Ad-EP4 after 28 days of femoral artery wire injury. The neointima area, intima-to-media (I/M) ratio and media area were analyzed. n = 4 per group. Black dotted line indicates internal and external elastic lamina. Between the black dotted line is media area. Scale bar = 100 μm. n = 4 per group. Data are presented as mean ± SEM. *$p < 0.05$, **$p < 0.01$.

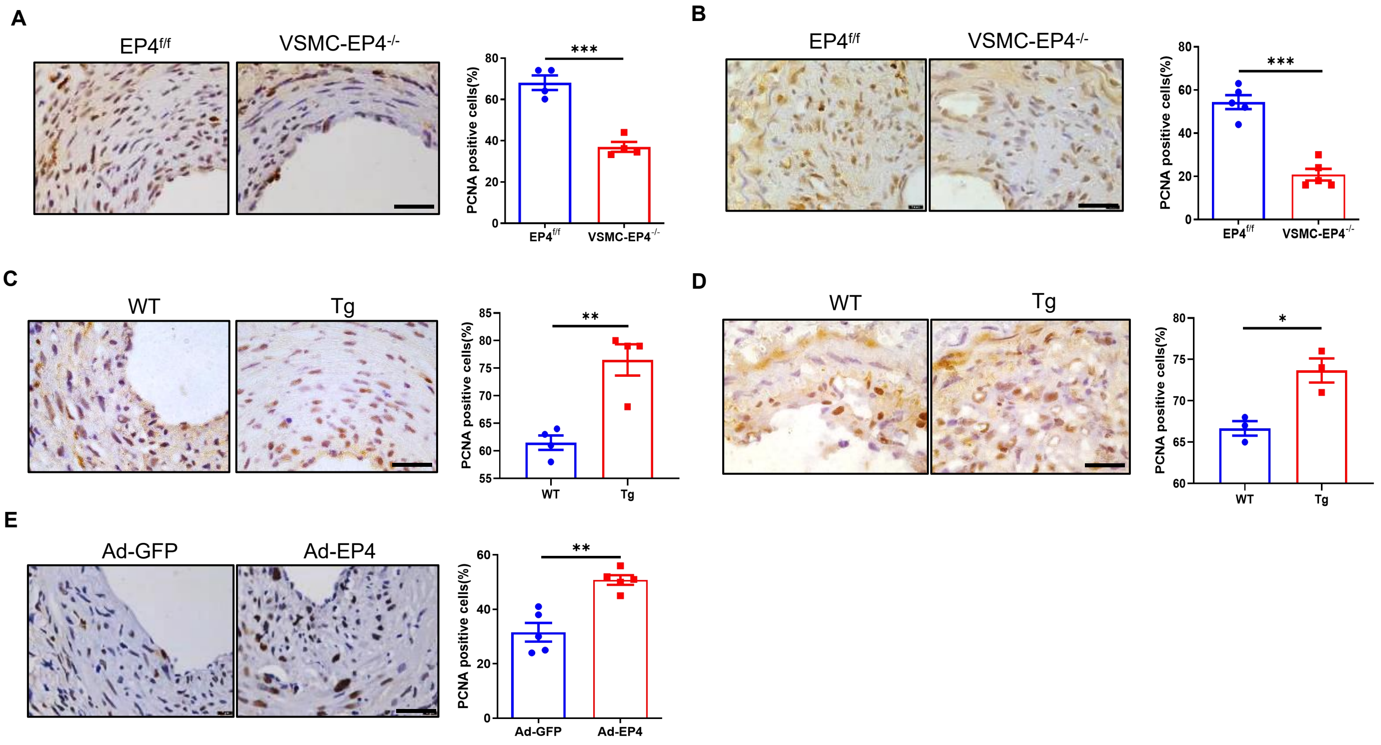

Figure S3. VSMC EP4 upregulates vascular PCNA expression in vivo.

A&B. The VSMC-EP4$^{-/-}$ and EP4f/f control mice were subjected to femoral artery wire injury (A) or carotid artery ligation (B) for 28 days. PCNA protein expression level was examined by immunohistochemistry staining. The percentage of PCNA positive cells was counted.

C&D. The VSMC-hEP4 Tg (Tg) and WT control mice were subjected to femoral artery wire injury (C) or carotid artery ligation (D) for 28 days. PCNA protein expression level was examined by immunohistochemistry staining. The percentage of PCNA positive cells was counted.

E. WT mice were subjected to femoral artery wire injury and the injured vessels were infected with the Ad-GFP or Ad-EP4 for 14 days. PCNA protein expression level was examined by immunohistochemistry staining. The percentage of PCNA positive cells was counted.

Scale bars = 25 μm. Data are presented as mean ± SEM. n = 4-5. *$p < 0.05$, **$p < 0.01$, ***$p < 0.001$.

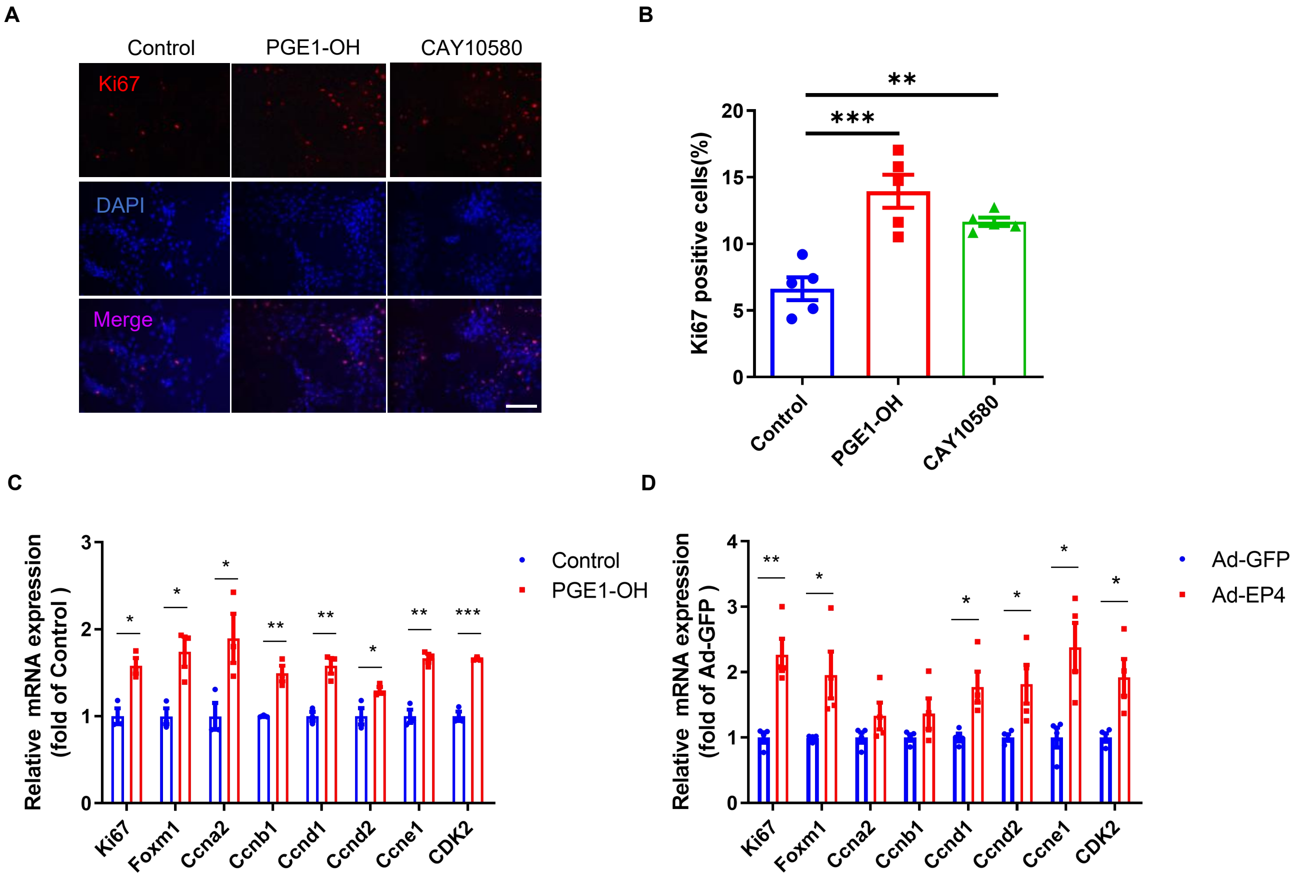

Figure S4. EP4 promotes the expression of genes and proteins involved in VSMC proliferation.

A&B. Rat VSMCs were treated with PGE1-OH (0.1 μM) or CAY10580 (1 μM) for 24 hours. Immunofluorescence analysis of Ki67 was performed and the quantification of Ki67 positive cells was showed in (**B**). Scale bars: 100 μm. n = 5.

C&D. RT-PCR analysis of the mRNA expression of proliferative genes including Ki67, Foxm1, Ccna2, Ccnb1, Ccnd1, Ccnd2, Ccne1 and CDK2 in VSMCs treated with PGE1-OH for 24 hours (**C**) and infected with the Ad-EP4 for 36 hours (**D**). n = 3-4.

Data are presented as mean ± SEM. **p* < 0.05, ***p* < 0.01, ****p* < 0.001.

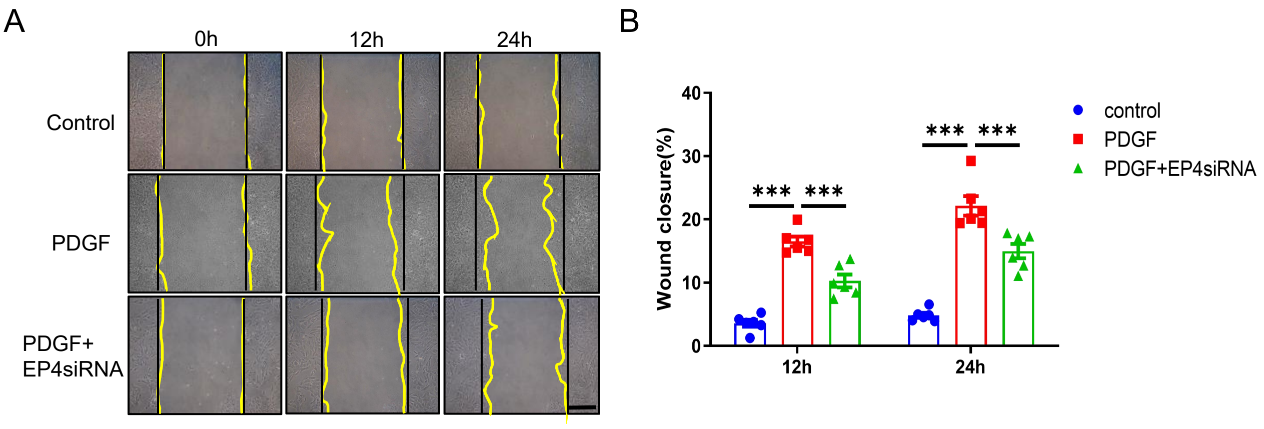

Figure S5. siRNA-mediated EP4 gene silencing blocks PDGF-BB-induced VSMC migration.

A&B. Knockdown EP4 by the siRNA-2 blocked the migration of VSMCs induced by PDGF-BB as assessed by a wound healing assay (**A**). The quantitative result was shown in (**B**). n = 6. Scale bars = 100 μm. Data are presented as mean ± SEM. ***$p < 0.001$.

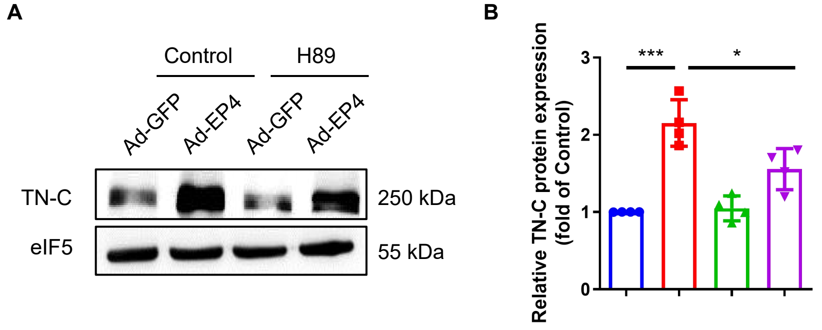

Figure S6. The PKA inhibitor H89 suppresses Ad-EP4-induced TN-C protein expression.

A&B. Rat VSMCs were pretreated with H89 (10 μM) for 30 minutes and then infected with the Ad-GFP or Ad-EP4 for 36 hours. TN-C protein expression was examined by western blot. n = 4. Data are presented as mean ± SEM. **p* < 0.05, ****p* < 0.001.

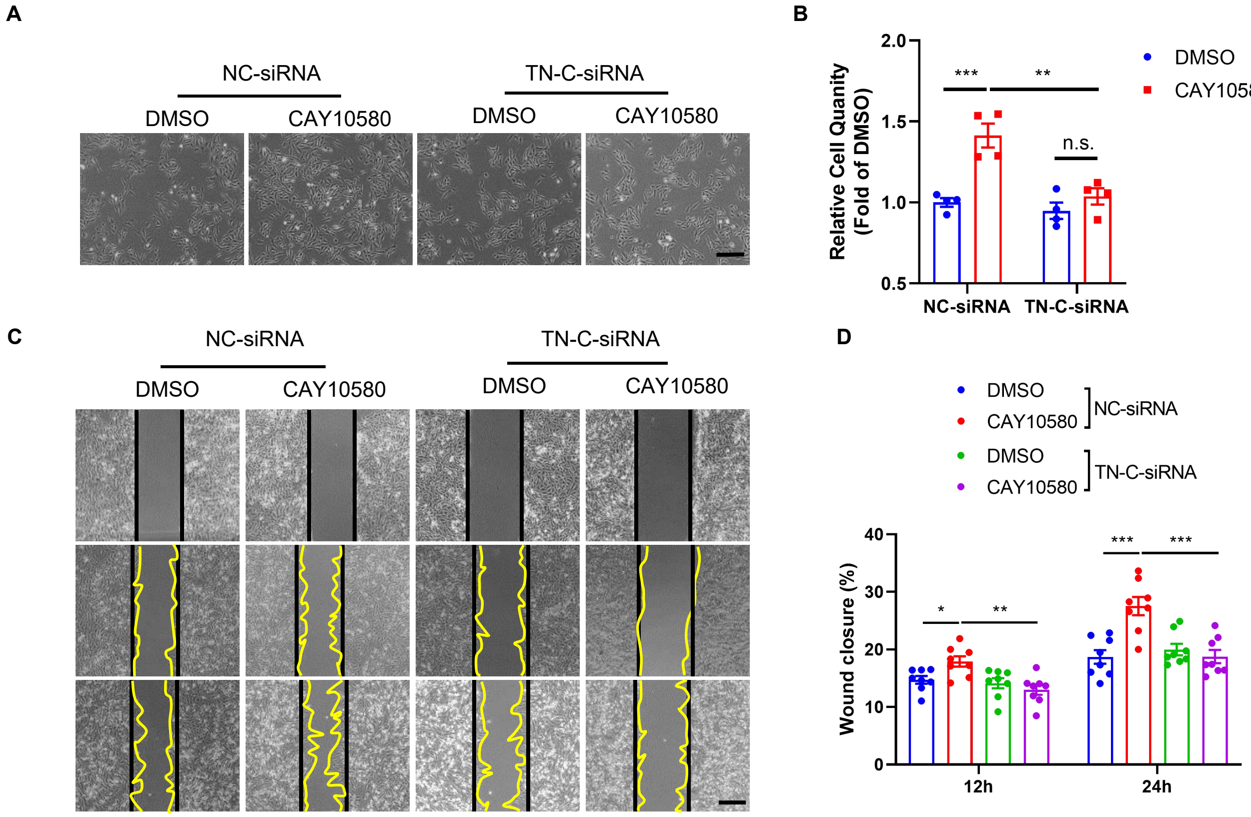

Figure S7. TN-C mediates CAY10580-elicited VSMC proliferation and migration.

A&B. Rat VSMCs were transfected with the TN-C siRNA-2 for 24 hours and then treated with CAY10580 (1 μM) for 24 hours. The cell numbers were counted at the end of experiment and relative cell quantity was presented in (**B**). n = 4.

C&D. Rat VSMCs were transfected with the TN-C siRNA-2 for 24 hours and then scratched. CAY10580 (1 μM) was treated and the images were taken under light microscope at indicated time point (12 and 24 hours) (**C**) and the closure rates were calculated (**D**). Scale bars: 100 μm. n = 8. Data are presented as mean ± SEM. *$p < 0.05$, **$p < 0.01$, ***$p < 0.001$.

Table S1. Primer pairs used for real-time PCR

Gene name	Forward primer sequence (5′-3′)	Reverse primer sequence (5′-3′)
Human-EP4	AGGCCATCCGAATTGCTTCT	GGCAGGAGGGTCTGAGATGT
Mus-EP1	TAACGATGGTCACGCGATGG	ATGCAGTAGTGGGCTTAGGG
Mus-EP2	ATGCTCCTGCTGCTTATCGT	AGGGCCTCTTAGGCTACTGC
Mus-EP3	GGATCATGTGTGTGCTGTCC	GCAGAACTTCCGAAGAAGGA
Mus-EP4	ATGGTCATCTTACTCATCGCCAC	CCTTCACCACGTTTGGCTGAT
Mus-GAPDH	CCTTCATTGACCTCAACTACATGGT	GAGGGGCCATCCACAGTCTTCTG
Rat-Ki67	AGGACTCGCAGTTTGAGAAGG	GTGGCTTTCAGAGAGCGTTG
Rat-Foxm1	CAGTTCGCGATCAACAGCAC	GAGCTTGTGGTAGGCCTGGT
Rat-Ccna2	GCTGACCAAGATCACCCACA	AACATCCCGCACGTCTGTAG
Rat-Ccnb1	AAACCCCTGCTGAGATCGAG	CCACAGGTTTTGGTAGGGCT
Rat-Ccnd1	TGGAGCCCCTGAAGAAGAG	AAGTGCGTTGTGCGGTAGC
Rat-Ccnd2	GCTGACCAAGATCACCCACA	AACATCCCGCACGTCTGTAG
Rat-Ccne1	CAAGTGGCCTACGTCAACGA	CCAGGACGCACAGGTCTAAA
Rat-CDK2	CTTAAGAAAATCCGGCTCGAC	ATCCAGCAGCTTGACGATGTTA
Rat-TN-C	CCACAGAAGCTGAACCGGAA	GGAGAACCCATGGCTGTTGT
Rat-GAPDH	GTCGGTGTGAACGGATTTGG	TACTCAGCACCAGCATCACC
